# *Twist-1* Up-Regulation in Carcinoma Correlates to Poor Survival

**DOI:** 10.3390/ijms151221621

**Published:** 2014-11-25

**Authors:** Alimujiang Wushou, Jing Hou, Ya-Jun Zhao, Zhi-Ming Shao

**Affiliations:** 1 Department of Breast Surgery, Fudan University Shanghai Cancer Center, Fudan University, No. 270 Dong An Road, Shanghai 200032, China; E-Mail: alimjan.wushur@gmail.com; 2 Department of Oncology, Shanghai Medical College, Fudan University, No. 270 Dong An Road, Shanghai 200032, China; E-Mail: 13671717421@163.com; 3 Department of Radiation Oncology, Fudan University Shanghai Cancer Center, Fudan University, No. 270 Dong An Road, Shanghai 200032, China; 4 Department Oral & Maxillofacial Surgery, College and Hospital of Stomatology, Xi’an Jiao Tong University, No. 98 Xiwu Road, Xi’an 710004, China; E-Mail: zhaoyajun_1@126.com

**Keywords:** *Twist-1*, immunohistochemistry, tumor, prognosis, meta-analysis

## Abstract

Epithelial-to-mesenchymal transition (EMT) facilitates tumor metastasis. *Twist* is a basic helix-loop-helix protein that modulates many target genes through E-box-responsive elements. There are two twist-like proteins, *Twist*-*1* and *Twist*-*2*, sharing high structural homology in mammals. *Twist-1* was found to be a key factor in the promotion of metastasis of cancer cells, and is known to induce EMT. *Twist-1* participation in carcinoma progression and metastasis has been reported in a variety of tumors. However, controversy exists concerning the correlation between *Twist-1* and prognostic value with respect to carcinoma. A systematic review and meta-analysis were performed to determine whether the expression of *Twist-1* was associated with the prognosis of carcinoma patients. This analysis included 17 studies: four studies evaluated lung cancer, three evaluated head and neck cancer, two evaluated breast cancer, two evaluated esophageal cancer, two evaluated liver cancer and one each evaluated osteosarcoma, bladder, cervical and ovarian cancer. A total of 2006 patients were enrolled in these studies, and the median trial sample size was 118 patients. *Twist*-*1* expression was associated with worse overall survival (OS) at both 3 years (hazard ratio “HR” for death = 2.13, 95% CI = 1.86 to 2.45, *p* < 0.001) and 5 years (HR for death = 2.01, 95% CI = 1.76 to 2.29, *p* < 0.001). Expression of *Twist-1* is associated with worse survival in carcinoma.

## 1. Introduction

Despite advances in understanding the pathogenesis, diagnosis, and new treatment approaches in cancer, the results are still unsatisfactory in most cases [1]. Cancer biomarkers can facilitate the early diagnosis and monitoring of the disease by contributing to our understanding of tumor biology and allowing more efficient therapeutic regimes to be applied earlier in the disease course, thus further improving patient survival [2]. The interpretation of protein expression of significant biomarkers is relatively simple in routine/diagnostic laboratories. Immunohistochemical staining of biomarkers is more promising for the evaluation of cancer risk. Thus, the identification of novel biomarkers that allow a more accurate prediction of treatment response and prognosis, ultimately leading to a favorable therapeutic outcome, is of paramount importance [3].

*Twist*, a highly conserved basic helix-loop-helix (bHLH) transcription factor, is characterized by a basic DNA binding domain that targets the consensus E-box sequence 59-CANNTG-39 and a helix-loop-helix domain. In mammals, two twist-like proteins, *Twist-1* and *Twist-2*, share high structural homology. The *N*-termini of *Twist-1* and *Twist-2* are more divergent, and *Twist-2* lacks a glycine-rich region that is present in *Twist-1* [4]. Epithelial to mesenchymal transition (EMT) is a novel cellular process that is essential for the development of metastatic disease. *Twist* has been identified as an inducer of EMT and a fundamental regulator of carcinoma metastasis [5,6].

*Twist-1* participation in metastasis has been reported in a variety of carcinoma. Dozens of studies have attempted to determine the prognostic value of *Twist-1* expression in carcinoma patients. However, controversy exists concerning the correlation between *Twist-1* and prognostic value with respect to carcinoma. Here, we performed a systematic review and meta-analysis in the published literature to clarify whether the expression of *Twist-1* was associated with the prognosis of carcinoma patients.

## 2. Results and Discussion

### 2.1. Description of Studies

**Figure 1:**
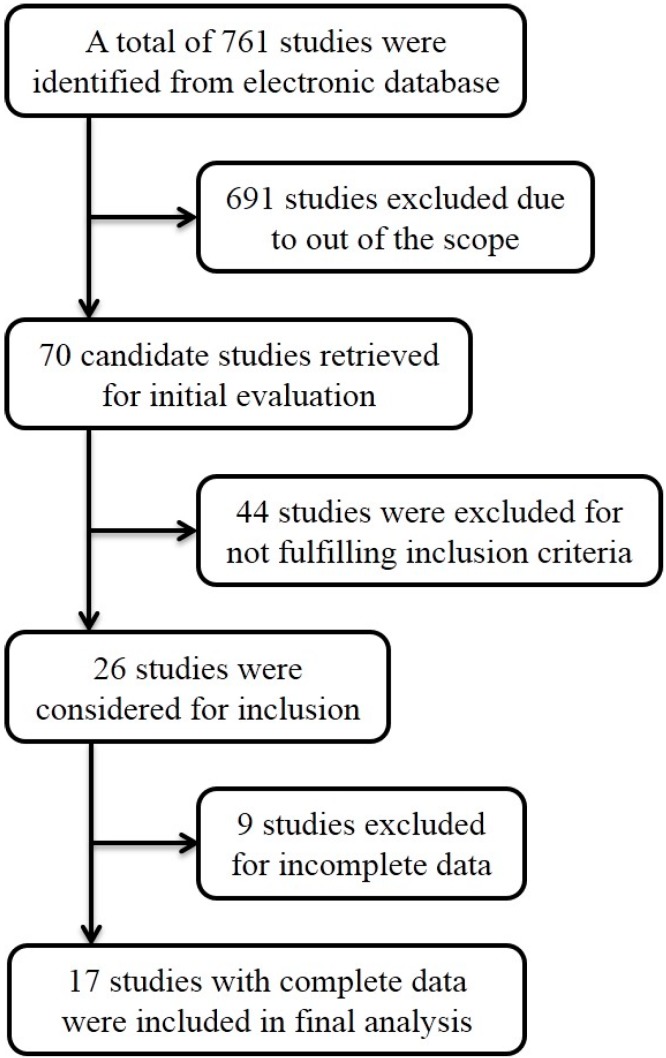
Flow diagram for selection of studies in the meta-analysis.

We identified 17 studies that used immunohistochemistry (IHC) techniques for the assessment of *Twist-1* expression [2,7,8,9,10,11,12,13,14,15,16,17,18,19,20,21,22] (Figure 1). Characteristics the of included studies are shown in Table 1. Four studies evaluated lung cancer, three evaluated head and neck cancer, two evaluated breast cancer, two evaluated esophageal cancer, two evaluated liver cancer and one each evaluated osteosarcoma, bladder, cervical and ovarian cancer. A total of 2006 patients were included in those studies, and the median trial sample size was 118 patients. The median follow-up of the 11 studies that reported follow-up times was 49.8 months (range = 22.7 to 117 months). All 17 studies reported data that allowed for the calculation of 3-year OS. Twelve studies presented data that allowed for assessment of 5-year OS.

**Table 1:** Baseline characteristics of the selected studies.

Author (Reference)	Year	Tumor Site	SS	*Twist* Positive	Antibody Used for IHC	Follow Time
Song LB *et al.* [8]	2006	Nasopharynx	75	44%	Goat polyclonal antibody, Santa Cruz, CA, USA	1–60 M
Kyo S *et al*. [7]	2006	Cervix	70	49%	*Twist* clone H-81, Santa Cruz, CA, USA	0.15–8.5 Y
Niu RF *et al.* [9]	2007	Liver	40	55%	Rabbit monoclonal antibody, Santa Cruz, CA, USA	0.15–4 Y
Hosono S *et al.* [2]	2007	Ovarian	82	40.2%	Rabbit polyclonal antibody, Santa Cruz, CA, USA	6–513 M
Fondrevelle ME *et al.* [10]	2009	Bladder	70	40%	*Twist* clone H-81, 1:50, Santa Cruz, CA, USA	1–89 M
Sasaki K *et al.* [12]	2009	Esophagus	166	42%	*Twist* clone H-8, Santa Cruz, CA, USA	1–181 M
Hung JJ *et al.* [11]	2009	Lung	87	36.8%	Rabbit polyclonal antibo dy ab50581, Abcam, UK	1–50 M
Zhao XL *et al.* [13]	2011	Liver	97	52.6%	Santa Cruz, CA, USA	NR
Soini Y *et al.* [17]	2011	Breast	388	47.7%	Mouse monoclonal antibody ab50887, Abcam, UK	NR
Lee KW *et al.* [16]	2011	Esophagus	165	51.1%	Mouse monoclonal antibody ab50887, Abcam, UK	2–155 M
Jouppila M A *et al*. [15]	2011	Pharynx	109	27%	Mouse monoclonal antibody, Abcam, UK	NR
Jiang W *et al.* [14]	2012	Lung	137	38%	Mouse monoclonal antibody ab50887, Abcam, UK	2–54 M
Wushou A *et al.* [19]	2012	Oral cavity	60	70%	Rabbit polyclonal antibody ab50581, Abcam, UK	6–59 M
Pallier K *et al.* [18]	2012	Lung	33	36.4%	Abcam, UK	NR
Yin K *et al.* [20]	2012	Osteosarcoma	107	31.8%	Rabbit monoclonal, R & D Systems, Minneapolis, MN, USA	2–100 M
Zhao M *et al.* [22]	2013	Breast	126	75.5%	Polyclonal antibody, Santa Cruz, CA, USA	NR
Hui L *et al.* [21]	2013	Lung	120	38.3%	*Twist* clone H-81, Santa Cruz, CA, USA	3–72 M

*Abbreviations:* SS, sample size; IHC, immunohistochemistry; NR, not reported; M, months; Y, year.

### 2.2. Association of Twist-1 with Prognosis

The combined analysis of 17 studies showed that *Twist-1* expression was associated with worse 3-year OS (HR for death = 2.13, 95% CI = 1.86 to 2.45, *p* < 0.00001). A similar result was found in the 5-year OS (HR for death = 2.01, 95% CI = 1.76 to 2.29, *p* < 0.00001) in a combination of 12 studies (Table 2). In the sensitivity analysis, the influence of each study on the pooled HR was assessed by repeating the meta-analysis while omitting each study one at a time. 

**Figure 2:**
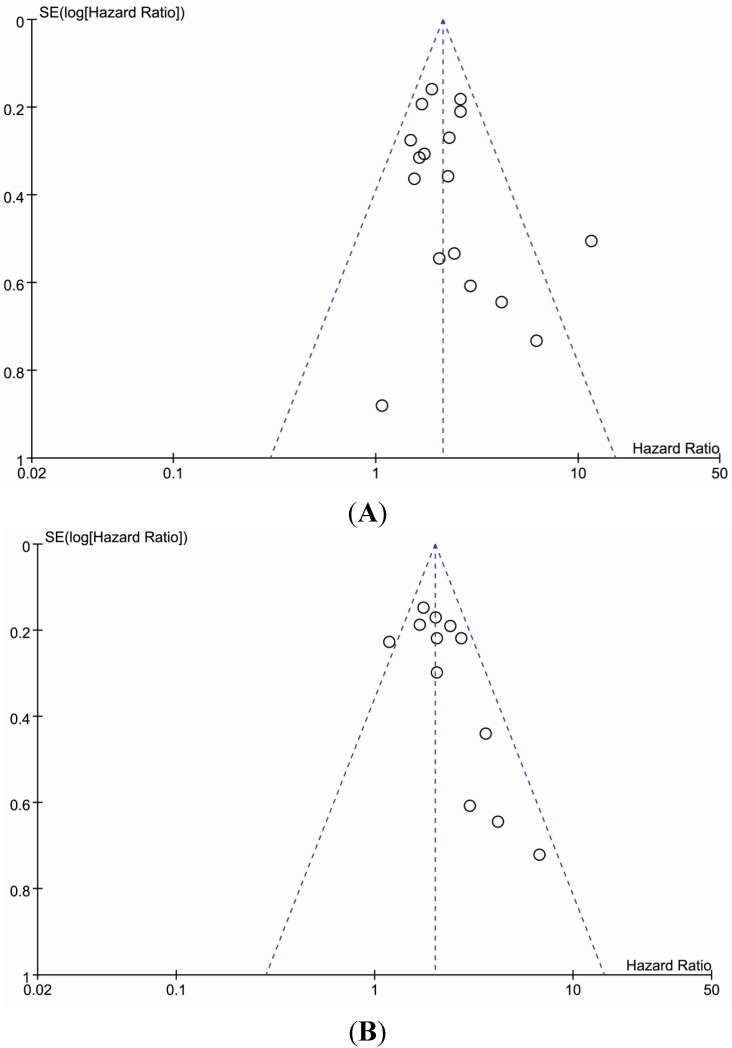
Funnel plot analysis to detect publication bias. Each point represents a separate study for the indicated association and the horizontal axes represent the hazard ratio with 95% confidence limits and vertical axes represent standard error of logarithmic hazard radio. (**A**,**B**) funnels plot for 3-year OS and 5-year OS, respectively.

Publication bias and sensitivity were analyzed in the included literature involving the overall HR estimation of 3 year OS and 5 years OS with tumor type (Figure 2A,B). The result showed that for the two breast cancer studies, heterogeneity was evident (*I*^2^ = 92%, *p* = 0.0004 and *I*^2^ = 94%, *p* < 0.00001, for 3-year and 5-year OS, respectively) (Table 3).

**Table 2:** The association between *Twist-1* expression and overall survival of carcinoma patients.

Studies for 3-Year Overall Survival	Weight (%)	HR 95% CI (Fixed Model )	Year
Song LB *et al.* [8]	3.8%	1.05 (0.52–2.14)	2006
Kyo S *et al.* [7]	1.4%	2.95 (0.90–9.67)	2006
Hosono S *et al.* [2]	5.0%	1.65 (0.89–3.07)	2007
Niu RF *et al.* [9]	5.3%	1.74 (0.95–3.18)	2007
Sakaki K *et al.* [12]	19.8%	1.88 (1.38–2.57)	2009
Fondrevelle ME *et al.* [10]	0.9%	6.22 (1.48–26.18)	2009
Hung JJ *et al.* [11]	3.9%	2.28 (1.13–4.60)	2009
Jouppila-matto A *et al.* [15]	13.3%	1.69 (1.15–2.47)	2011
Soini Y *et al.* [17]	2.0%	11.56 (4.30–31.08)	2011
Lee KW *et al.* [16]	14.9%	1.87 (1.31–2.68)	2011
Pallier K *et al.* [18]	1.2%	4.19 (1.18–14.86)	2012
Zhao XL *et al.* [13]	6.8%	2.29 (1.35–3.91)	2012
Yin K *et al.* [20]	1.7%	2.04 (0.70–5.95)	2012
Wushou A *et al.* [19]	0.6%	1.08 (0.19–6.07)	2012
Jiang W *et al.* [14]	1.7%	2.45 (0.86–7.00)	2012
Zhao M *et al.* [22]	6.5%	1.49 (0.86–2.56)	2013
Hui L *et al.* [21]	11.2%	2.62 (1.73–3.96)	2013
**Total (95% CI)**	**100.0%**	**2.13 (1.86–2.45)**	
**Heterogeneity: Chi^2^ = 24.75, df = 16 (*p* = 0.07); *I*^2^ = 35%; Test for overall effect: Z = 9.78 (*p* < 0.00001)**			
**Studies for 5-Year Overall Survival**	**Weight (%)**	**HR 95% CI (Fixed Model )**	**Year**
Kyo S *et al.* [7]	1.2%	3.00 (0.91–9.85)	2006
Hosono S *et al.* [2]	5.1%	2.05 (1.14–3.69)	2007
Fondrevelle ME *et al.* [10]	0.9%	6.80 (1.65–27.97)	2009
Sakaki K *et al.* [12]	20.4%	1.57 (1.17–2.10)	2009
Jouppila-matto A *et al.* [15]	12.8%	1.70 (1.17–2.46)	2011
Lee KW *et al*. [16]	15.5%	2.02 (1.44–2.52)	2011
Soini Y *et al.* [17]	9.4%	2.73 (4.30–31.08)	2011
Pallier K *et al.* [18]	1.1%	4.19 (1.18–14.79)	2012
Yin K *et al.* [20]	2.4%	3.60 (1.52–8.53)	2012
Zhao XL *et al.* [13]	9.5%	2.06 (1.34–3.17)	2012
Zhao M *et al.* [22]	8.8%	1.19 (0.76–1.85)	2013
Hui L *et al.* [21]	12.7%	2.42 (1.67–3.51)	2013
**Total (95% CI)**	**100.0%**	**2.01 (1.76–2.29)**	
**Heterogeneity: Chi^2^ = 18.00, df = 11 (*p* = 0.08); *I*^2^ = 39%; Test for overall effect: Z = 9.94 (*p* < 0.00001)**			

**Table 3:** Subgroup heterogeneity analysis of *Twist-1* expression in different carcinomas.

Cancer Type	Heterogeneity	Pooled HR (95% CI)	*p*-Value	Reference
Breast cancer	3-year OS: *I*^2^ = 92%, *p* = 0.0004	2.39 (1.49–3.85)	*p* < 0.001	[17,22]
	5-year OS: *I*^2^ = 94%, *p* < 0.00001	2.34 (1.72–3.20)	*p* < 0.001	
Lung cancer	3-year OS: *I*^2^ = 0%, *p* = 0.87	2.60 (1.18–3.61)	*p* < 0.0001	[11,14,18,21]
	5-year OS: *I*^2^ = 0%, *p* = 0.41	2.52 (1.77–3.61)	*p* < 0.0001	
Liver cancer	3-year OS: *I*^2^ = 0%, *p* = 0.50	2.03 (1.36–3.03)	*p* = 0.0005	[9,13]
Head and neck cancer	3-year OS: *I*^2^ = 0%, *p* = 0.48	1.50 (1.08–2.08)	*p* = 0.02	[8,15,19]
Esophagus cancer	3-year OS: *I*^2^ = 46%, *p* = 0.17	2.17 (1.72–2.75)	*p* < 0.00001	[12,16]
	3-year OS: *I*^2^ = 75%, *p* = 0.05	2.09 (1.67–2.61)	*p* < 0.00001	

### 2.3. Discussion

*Twist* is overexpressed in various cancers [12,17,19]. Most importantly, *Twist-1* and related signal transduction pathways play important roles in carcinoma progression and may serve as targets for treating carcinoma [23]. The current interest in *Twist-1* as a potential prognostic marker for carcinoma stems from the fact that many experimental studies have linked *Twist-1* expression with worse survival in carcinomas such as esophageal, oral, lung, ovarian and cervical cancer [2,7,11,16,19]. Despite the fact that the association of *Twist-1* expression with tumor metastatic process has been explored for several years, controversy remains and the available data have not been comprehensively analyzed. In this study, we meta-analyzed the published data concerning the expression of *Twist-1* in carcinomas and their association with survival for studies that evaluated *Twist-1* by IHC. Pooled HR results show that expression of *Twist-1* was associated with worse survival. These results can be observed at both 3 and 5 years. In other words, summary estimates support the hypothesis that *Twist-1* expression is associated with worse survival in carcinomas, which is, *Twist-1* may serve as a prognostic marker for carcinomas. 

Biomarkers can be detected in cancerous tissue, blood, and body fluids. Cancerous tissue samples examined by IHC are preferred for the evaluation of tumor markers. Studies, measuring *Twist-1* gene or mRNA level by PCR, were not included in this meta-analysis. Few studies demonstrate correlations between the *Twist-1* gene or mRNA expression and prognosis. We selected only studies that evaluated *Twist-1* by IHC because there was consistency in the evaluation process among studies. It is usually required to make a cutoff value to evaluate immunoreactivity of any protein expression in tissue specimens. Thus, subjective cut-off values in each study affected the overall data on the study of *Twist* expression in solid tumors. The results of this meta-analysis should be interpreted very cautiously; the majority of published studies did not disclose the information on patients preoperative or postoperative treatment or the type of adjuvant therapy each patient received, which may also affect the prognosis of the patients. All of this insufficient information could contribute to additional inconsistencies and creation of potential selection bias. Thus, our current data need to be substantiated by adequate prospective studies.

Although the present meta-analysis has some advantages over other individual studies, a few limitations were also inherent. First, because this is a literature-based analysis, it is compromised by the potential for publication bias, whereby predominantly positive results were published, thus inflating our estimate for the association between *Twist-1* and poor outcome. Second, there is no accepted and validated method for assessment of *Twist-1* expression. Most of the studies used the two-way scoring system for immunostaining evaluation; meanwhile some papers were using one-way scoring considering only the proportion of positive cells ignoring the staining intensity. Therefore, there may be underlying heterogeneity. An internationally accepted and validated method for *Twist-1* testing is warranted. Third, the studies included in the meta-analysis were from different sources of *Twist-1* antibody and dilutions of the antibodies, indicating a possibility that antibody factors can confound the results. In addition, experimental processes may partly influence the significance of the clinicopathological outcome in survival analyses and partially account for the inter-study heterogeneity.

## 3. Experimental Section

### 3.1. Identification and Selection of Studies

The PubMed, ISI Web of Science, and Embase databases were searched for studies evaluating the expression of *Twist* and survival in solid tumors. The search ended on 31 May 2014, and no lower date limit was used. We used the medical subject heading terms “*Twist* and cancer” and limited the results to human studies. In addition, we used the entry “*Twist*” and the name of each specific solid tumor (for example *Twist* and breast cancer) to recognize additional studies. Eligibility criteria were the measurement of *Twist-1* by immunohistochemistry (IHC), availability of survival data for at least 3 years, and publication in English. Studies evaluating gene expression of *Twist-1* measured by polymerase chain reaction or fluorescence *in situ* hybridization were excluded from the analyses. Citation lists of retrieved articles were manually screened to ensure sensitivity of the search strategy. Study selection was based on the association of *Twist-1* and survival.

### 3.2. Data Collections and Analysis

The suitability of studies for inclusion was independently assessed by two authors and any lack of clarity or disagreement was resolved through discussion. We developed a data extraction sheet based on the Cochrane Consumers and Communication Review Group’s data extraction template. The following details were extracted: first author, year of publication, tumor type, number of patients, follow-up time and antibody used for the evaluation, method and score for its assessment, and cut-off for considering *Twist-1* as a positive expression. Outcomes of interest was for three- and five-year overall survival (OS). 

For each study, HR was estimated using an approach reported by Parmar *et al*. [6].The most accurate approach is to obtain the HR estimate and 95% CI directly from the paper, or calculating them using the parameters such as the O-E statistic and variance offered in the manuscript. Otherwise, the number of patients at risk in each group, the number of events and *p*-value of the log-rank statistic was retrieved to permit an approximate calculation of the HR estimate and its variance. If the study did not provide a HR but reported the data in the form of the survival curve, survival rates at certain specified times were extracted from them for the reconstruction of the HR estimate and its variance, with the assumption that the rate of patients censored was constant during the follow-up.

### 3.3. Statistical Analysis

Kaplan-Meier curves were interpreted by the Engauge Digitizer version 4.1 (free software downloaded from http://sourceforge.net, Dice Holdings, Inc., New York, NY, USA). Data combining were performed by RevMan version 5.2 (free software downloaded from http://www.cochrane.org, The Cochrane Collaboration, The Nordic Cochrane Centre, Copenhagen, Denmark, 2012). The combined HR with 95% CI was utilized to calculate and assess the strength of the association of *Twist-1* expression. An observed HR > 1 indicated a poor prognosis for the group with *Twist-1* positive expression and would be considered to be statistically significant if the 95% CI did not overlap 1. Estimates of HR were weighted and pooled utilizing the fixed-effect model. Heterogeneity was assessed by inspection of the forest plot, Cochran chi-squared test, and the *I*^2^ statistical percentage. Sensitivity analysis and potential publication bias were also evaluated. All statistical tests were two-sided, and statistical significance was defined as *p* less than 0.5.

## 4. Conclusions

In conclusion, our analyses show that expression of *Twist-1*, as measured by IHC, is associated with a worse prognosis in carcinoma, which suggests that the development of strategies against this transcription factor could be a reasonable therapeutic approach. However, the potential of *Twist-1* as a therapeutic target in cancer treatment still requires validation in further multicenter, longitudinal, prospective, large cohort studies. 
